# Effects of Waste Frying Oil and Crumb Rubber on the Characteristics of a Reclaimed Asphalt Pavement Binder

**DOI:** 10.3390/ma14133482

**Published:** 2021-06-23

**Authors:** Munder Bilema, Mohamad Yusri Aman, Norhidayah Abdul Hassan, Zaid Al-Saffar, Nuha S. Mashaan, Zubair Ahmed Memon, Abdalrhman Milad, Nur Izzi Md Yusoff

**Affiliations:** 1 Department of Highway and Traffic Engineering, Faculty of Civil Engineering and Environmental, Univesiti Tun Hussein Onn Malaysia, Parit Raja 86400, Johor, Malaysia; mdyusri@uthm.edu.my; 2 Department of Geotechnics & Transportation, School of Civil Engineering, Faculty of Engineering, Universiti Teknologi Malaysia, Johor Bahru 81310, Johor, Malaysia; hnorhidayah@utm.my; 3 Department of Building and Construction Engineering, Technical College of Mosul, Northern Technical University, Mosul 41001, Iraq; zaid.alsaffar025@gmail.com; 4 School of Civil and Mechanical Engineering, Faculty of Science and Engineering, Curtin University, Perth 6102, Australia; Nuhas.Mashaan1@curtin.edu.au; 5 Department of Engineering Management, College of Engineering, Prince Sultan University (PSU), Riyadh 11586, Saudi Arabia; zamemon@psu.edu.sa; 6 Department of Civil Engineering, Universiti Kebangsaan Malaysia, Bangi 43600, Selangor, Malaysia; miladabdalrhman@siswa.ukm.edu.my

**Keywords:** reclaimed asphalt pavement (RAP), crumb rubber (CR), waste frying Oil (WFO), RAP binder (RAPB)

## Abstract

The reclaimed asphalt pavement (RAP) has become a moderately common practice in most countries; Hence, rejuvenating materials with RAP have earned publicity in the asphalt manufacturers, mainly due to the increasing raw material costs. In this study, the crumb rubber (CR) and waste frying oil (WFO) utilized as waste materials to restore the properties and enhance the rutting resistance of the RAP. Several physical, rheological, chemical properties of bituminous binders were tested. The result showed that the RAP bituminous binders incorporating WFO and CR decreased softening points and the increased penetration value; these translate to an increase in penetration index. Moreover, the viscosity of the WFO/CR combination reclaimed asphalt pavement binder showed better workability and stiffness, as well as a low storage stability temperature (less than 2.2 °C) with an acceptable loss upon heating. Without chemical reaction was observed between the waste-frying oil with the rubberized binder and the reclaimed asphalt pavement binder. Additionally, the WFO/CR rheological properties combined with the reclaimed asphalt pavement binder were comparable to the control sample. The incorporation of CR with WFO as a hybrid rejuvenator enhanced the rutting resistance. Therefore, the presence of WFO/CR has a considerable influence on the RAP binder properties while preserving a better environment and reducing pollution by reusing waste materials.

## 1. Introduction

Recently, reclaimed asphalt pavement (RAP) has gained popularity in the production of asphalt mixtures and has become a cost-effective method of pavement construction and rehabilitation. RAP is beneficial from the technical, economical, and environmental perspectives. The age of the asphalt mixture is usually between 7–15 years, but numerous asphalt mixtures require major servicing less than 10 years after construction [1,2]. Throughout the service life of the asphalt pavement, bituminous binder suffers from environmental conditions and traffic overload that tend to make an adequate binder viscoelastic, severely oxidized, and increasingly aged [3]. These factors directly influence the behavior of the asphalt pavement. RAP will produce stiffer binders that may cause workability issues and decrease asphalt pavements’ quality [4]. To enhance the bituminous binder rheology, waste cooking oil (WCO) could be utilized as a rejuvenator in binder via a low-viscosity oil content to assortment its chemical structure and ratio to manufacture a bituminous binder. The use of waste oil as a rejuvenator is widely used; therefore, a viable alternative in mitigating this problem [5,6].

The determination criteria of a bituminous binder rejuvenator must comply and cover an adequate capacity of RAP material, get in the aged binder, and produce a more robust and coherent batch to achieve the same level as the conventional asphalt mixture. Asphalt mixtures containing 15% of RAP or less perform similarly, if not better than conventional asphalt materials in terms of indirect tensile strength, moisture susceptibility, permanent deformation, and fatigue [7,8]. Reusing reclaimed asphalt can increase binder rheology behavior by improving its viscosity and increasing stiffness to enhance pavement performance. In the blending process, the virgin binder is supposed to rejuvenate the RAP binder to produce the target viscosity [9]. However, when RAP binder and virgin binder blends, both in asphalt plants and during pavement service life, change the chemical structure and formation concerning the functional groups in the bituminous binder. This is of great concern since the RAP binder is known to be readily oxidized by aging, becoming increasingly brittle. Subsequently, it becomes less adhesive but more cohesive, being prone to lose its binding capacity. The chemical and physical properties of the binder are aging due to oxidation changes in the structural and functional grouping that influences the binder-aggregate interaction. There is a correlation between the performance behavior of asphalt mixtures and the chemical composition of the bituminous binder [10,11]. The bituminous binder comprises four fractions: asphaltenes, aromatics, resins, and saturates. Upon binder aging, the asphaltene and resin content increases while the saturates content decreases. Additionally, there increases in viscosity and softening point properties after the aging process, consequently lowering the ductility and penetration relative to the virgin binder. Thus, aging reduces the bitumen performance and produces a weak condition that influences the service life of the pavement and subsequently, increases the maintenance cost [12,13].

Waste frying oil (WFO) is a kind of obtainable material for further reused since it can become an environmental pollutant due to elimination issues and the potential pollution of water resources. Recently, numerous studies have been performed to investigate the use of WFO in RAP [14,15,16]. García et al. [17] examined the characteristics of asphalt mixture containing sunflower capsules; they summarised that the sunflower oil capsules reduced the indirect tensile strength and stiffness modulus of the asphalt mixture. Wen et al. [18] performed experiment research to investigate waste cooking oil as an alternative waste resource to increase asphalt mixture performance, they concluded that the physical characteristics of WFO could impact the performance of the reclaimed asphalt pavement binder. Some researchers indicated that waste cooking oil enhances bitumen characteristics and increases asphalt pavement quality [19].

Increasing bitumen stiffness and getting appropriate asphalt workability at high temperatures are desirable in asphalt mixtures. Several modifiers improve some properties of asphalt mixtures, such as crumb rubber (CR) [20]. CR is one alternative for improving asphalt mixtures by creating stiffer mixtures at high service temperatures [21]. In contrast, the utilization of CR in the asphalt pavement industry is considered an alternative solution to reduce the issue of polluting the environment while improving the properties of the asphalt mixture [22,23]. However, the use of CR has a considerable influence on the bituminous binder and can modify the behavior of the asphalt pavement from soft to hard, decrease the penetration, and raise the softening point. Using CR in asphalt mixing, the landfill disposal problem of automobile tires as waste rubber tires can be reduced. [24,25]. Serval studies concluded that the addition of the CR leads to improving the rutting resistance, indirect tensile strength, and resilient modulus [26,27,28].

Based on the literature mentioned above, a few studies emphasized WFO and CR as hybrid rejuvenators to restore the RAP binder’s properties and avoid the rutting issue caused by adding WFO. Therefore, the current study was undertaken to evaluate the RAP binder’s physical, rheological, and chemical attributes with and without the hybrid rejuvenator. There are two reasons for adding RAP and WFO, together with CR, in asphalt mixture, to lower the use of natural resources and discover a sustainable pavement substitutional.

## 2. Experimental Design

### 2.1. Materials

The conventional 60/70 penetration grade bitumen provided by Kemaman Bitumen Company Sdn. Bhd Selangor, Malaysia, was used as the base binder with density and flash point equal to 1.02 gm/cm³ and 240 °C, respectively. The RAP was collected from the PLUS Highway Company Johor, Malaysia, RAP was milled from the North-South Expressway E2 at point 140 km to 148 km. Subsequently, the RAP was exposed to the extract-and-recover process to extract the reclaimed asphalt pavement binder for the binder experiments. According to ASTM D2172, RAP binders were extracted from the reclaimed asphalt aggregates. In this extraction and recovery process, the Trichloroethylene chloride was used to extract the binder from reclaimed asphalt pavement by mixing trichloroethylene chloride with reclaimed asphalt pavement then heated until Trichloroethylene chloride evaporates from the mixture. According to ASTM D5404, the recovery process of RAP was performed using a rotary evaporator, where RAP binders were separated from the solvent. Following that, the penetration test was conducted to determine the grade of RAP binders. The WFO was collected from three different restaurants in Batu Pahat, Johor, Malaysia. The WFO’s was exposed to a simple filtering process using filtration paper with a diameter of 150 mm. Nordiana et al. [29] reported that treated oil could enhance the rheological characteristics of the aged binder. The CR, retained in a 0.15 mm sieve (mesh 40) by the Miroad Rubber Industry, Johor, Malaysia, was used. This study conducted the penetration, softening point, ductility, and viscosity tests to determine the WFO and CR contents [30].

### 2.2. Mixing Method

The current research utilized 60/70 penetration grade bitumen and various percentages of RAP, WFO, and 1.5% of CR as additives. The RAPB and virgin binders were blended at 160 °C for 20 min. The 25%RAPB+WFO+CR and 40%RAPB+WFO+CR binders were produced according to the following steps. First, the virgin and aged binders were heated at 110 and 150 °C, respectively, for 1 h and then poured in a container and placed on the hot plate. Subsequently, 1.5% of CR was added into the binder at 177 °C for 20 min [31,32]. Then, the hot plate temperature was adjusted to 150 °C for the next additive; 5 min was provided to allow the hot plate to reach the target temperature. Then, the required amount of WFO was added at 150 °C for 20 min to make the binder fluid enough and to ensure uniformity and homogeneity [33]. Finally, the modified binder was poured into containers to be used for the following testing.

### 2.3. Laboratory Tests

The performance of asphalt mixtures can be similar to their material properties. In this research, the control sample was distinguished by its consistency or capability to flow at various temperatures, viscosity, chemical structure, and formation. The physical, rheological, and chemical tests of bituminous binders were carried out according to standard specifications for the road work, as shown in Table 1.

## 3. Results and Discussion

### 3.1. Characteristics of the Waste Frying Oil

#### 3.1.1. Physical Properties of the Waste Frying Oil

The physical properties of WFO’s were tested in the fuel laboratory, Department of Mechanical, Universiti Tun Hussein Onn Malaysia. Table 2 shows the physical properties of the WFO’s collected from the local restaurants. The three sources of WFO were processed from palm oil. The result indicates that Source 2 of WFO shows a high value of specific gravity, moisture content, and heat loss but low in flash point and viscosity. The flashpoint result for oil was a vital characteristic to recover the RAP binder because when the oil is blended with a binder, it requires a high mixing temperature up to 165 °C. The acceptable flash point result for rejuvenators must be more than 180 °C to satisfy the safety requirements in the construction area. The Source 2 oil sample transformed into wax with the increased temperature, and the test was terminated at 110 °C. Also, the low flashpoint temperature result for Source 3, which could be related to the oil manufacture quality or the frying process. On the other hand, the result of a flashpoint for the source 1 oil sample was found suitable to be utilized as a rejuvenator in the RAP binder.

The heat loss test measures the loss in mass of oil after exposure to high pressure and temperature. Even after the filtration process, small particles remain in the oil, leading to a lack of quality. Oil from Sources 2 and 3 are inadequate for the use with asphalt binder due to their high percentages of loss in weight as well as low viscosity compared to oil from Source 1.

#### 3.1.2. Fatty Acids of the Waste Frying Oil

The chemical fatty acids in the WFO were performed using gas chromatography-mass spectrometry (GCMS). Table 3 shows the fatty acids of the waste frying oil. From Table 3, it can be concluded that WFO consists of Oleic acid (54.81%), Linoleic acid (11.38%), and Palmitoleic acid (8.92%). The high content of the linoleic acids affects the surface coating. It leads to strong cohesion with the aggregate, which is agreed by the previous study [34]. Moreover, with the high amount of Oleic acid and Palmitoleic acid, it can be split by thermal cracking or catalytic cracking for the potential format of the hydrocarbon chain [35].

#### 3.1.3. Chemical Groups of Waste Frying Oil

**Figure 1:**
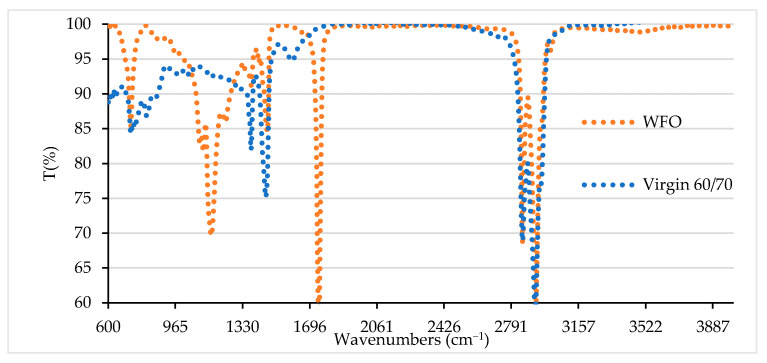
Chemical group absorption peaks in the WFO and bitumen.

The chemical structure and formation were assessed using Fourier Transforms Infrared Spectroscopy (FTIR). Figure 1 displays the result of FTIR for the WFO and virgin binder. This figure shows absorption peaks at 3006, 2922, and 2853 cm^−1^ corresponding to carboxylic acids (O-H); 1743 cm^−1^ corresponding to carbonyl (C꓿O); 1464 cm^−1^ corresponding to alkanes (CH₂); 1377 cm^−1^ corresponding to alkanes (CH₃); 1230 cm^−1^ corresponding to alkyl ketone (C-N); 1159 cm^−1^ corresponding to polysaccharide (C-O-C); 1116 cm^−1^ corresponding to esters (C-C); 1097 cm^−1^ corresponding to carbohydrate (C-O); and 72 cm^−1^ corresponding to the fingerprint region (C-H). Therefore, evaluation of the FTIR for the WFO revealed no change compared with bitumen except the slight peak near 721 cm^−1^ which represents the aromatic band [36]. The WFO has no absorption peaks near 800 cm^−1^ or 1588 cm^−1^, which represent the benzene bands. As a result, harmful gases will potentially be reduced compared to virgin binder during the pavement construction process, providing a friendly and healthy environment for construction workers.

### 3.2. Physical and Chemical Properties of Bituminous Binders

#### 3.2.1. Penetration

**Figure 2:**
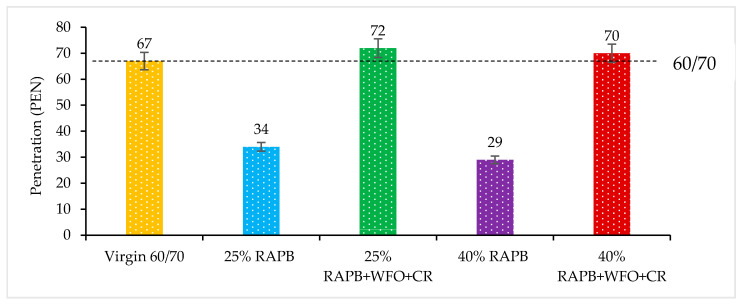
Penetration results at 25 °C for all binders.

Figure 2 illustrates the penetration result at 25 °C for all binders. As seen in Figure 2, with 25 and 40% of reclaimed asphalt pavement binder (RAPB) content in the binder, respectively, it can produce a stiffer binder and increase the penetration values with increasing the RAPB. Also, lower penetration values display in binders with 25 and 40% of RAPB content compared to the control sample, related to oxidation aging during the service life of the asphalt pavement. The aged binder extracted from RAP contains a high percentage of asphaltene, which leads to increased stiffness of the binder [37]. The binder with 25 and 40% of RAPB content gives 34 PEN and 29 PEN, respectively. As stated by Zargar et al. [14], the decrement in the maltenes to asphaltenes ratio in RAPB is a result of an increase in penetration value.

On the other hand, the binders with 25 and 40% of RAPB content incorporating WFO/CR exhibited comparable penetration values to the virgin binder. A high amount of oil reduces the hardness of bitumen and the dosage of small CR, balancing the softness of the binder. Also, incorporating the CR with WFO into aged binders content 25 and 40% of RAPB gives penetration values 72 PEN and 70 PEN, respectively. It determined that the penetration outcome will increase linearly as the quantity of additional WFO into the RAP binder increased. These outcomes are similar to those of a previous study [19].

Table 4 displays the significant differences of bituminous binders for the penetration results. From the paired t-test result shown in Table 4, there is a significant difference between bitumen and RAPB with (*p* ≤ 0.05) indicating that the addition of RAPB has a considerable effect on the stiffness of the binder. On the other hand, there is no significant difference between bitumen and the RAPB with *p* = 0.341 and *p* = 0.499, respectively. The analysis showed that the ability of WCO and CR to recover the properties of RAPB.

#### 3.2.2. Softening Point

**Figure 3:**
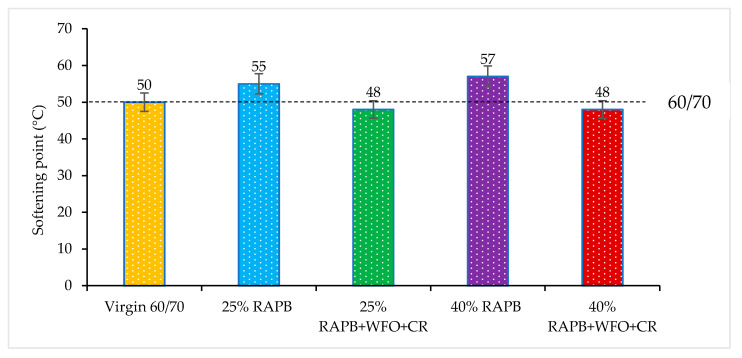
Softening point result for all binders.

The outcome of the softening point test is shown in Figure 3. The softening point shows the temperature degree that the binder transfers from a semi-solid state to a soft status. The result demonstrates that bituminous binders with 25 and 40% RAPB content show the highest softening point values with 55 and 57 °C, respectively. This due to the hardness of the aged binder; increased binder hardness requires higher temperatures to soften the binder. Also, asphaltene with a higher molecular weight produces a harder binder as well. These findings are consistent with a previous study [38].

In contrast, the combination of WFO and CR has a positive effect on the softening point. As shown in the results of the bituminous binders with 25 and 40% of RAPB content incorporating WFO/CR, both reaching the softening point value of 48 °C, this is the proper softening point value for a conventional mixture with virgin bitumen. The binder with 25 and 40% of RAPB content incorporating the combination of WFO and CR gives softening point results comparable to the virgin bitumen. It is related to the significant effect and the WFO’s ability of WFO to soften the aged binder [12].

The mean results were evaluated using a paired t-test to compare the significant differences of all binders with virgin bitumen. Table 5 displays a comparison of the significant differences between virgin bitumen, RAP, and RAPB. There is a significant difference between the virgin bitumen and binder containing 25 and 40% RAPB with *p* = 0.028 and *p* = 0.037, respectively. The analysis showed that minor significant differences between the virgin bitumen and binder contain 25 and 40% RAPB because both *p* -values were close to 0.05. Meanwhile, there is no significant difference between the virgin bitumen and RAPB with WFO and CR with (*p* > 0.05). It concluded that the addition of WFO and CR reduced the softening point temperature when it was added to the RAPB.

#### 3.2.3. Penetration Index

**Figure 4:**
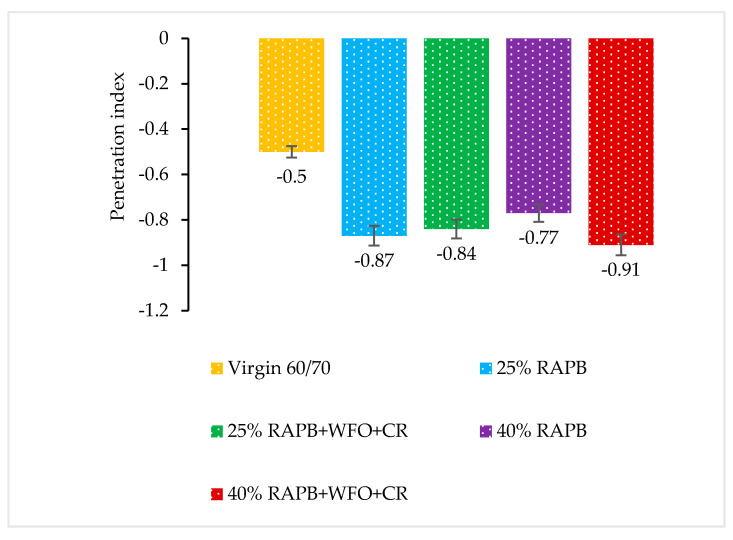
PI outcome for all binders.

Penetration index (PI) was obtained to classified the type of bituminous binder. The penetration index needs two variables, which are softening point and penetration, to calculate the PI [39]. This characteristic was able to determine the suitability of the bitumen to be used in the highway construction industry. Figure 4 illustrates the PI result for various Binders. 

It can be observed that the result of all binders was within the range for the conventional paving binder, which is between −2 to 2. Besides, the binders with 25 and 40% RAPB content significantly affect the PI result, which makes the binder less temperature susceptible to the other binders. Also, the binders with 25 and 40% of RAPB content incorporating the combination of WFO and CR have a comparable result as the virgin bitumen because the combination of WFO and CR gives the balance to the binder. As a result, all the binders consider as conventional paving binders concerning the temperature susceptible value of each binder [40].

The PI results were analyzed using paired sample t-test to compare the significance of group means between the virgin bitumen and RAPB. Table 6 listed the significant differences in the PI results. There were statistically significant differences between virgin bitumen and all binders with (*p* ≤ 0.05); however, the significant differences were major for the binder contain a RAPB while the significant differences were minor for the binder contain WFO and CR.

#### 3.2.4. Storage Stability

**Figure 5:**
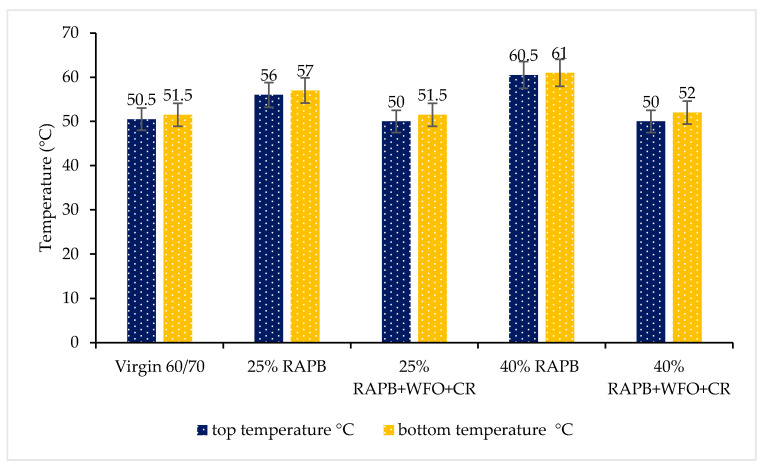
Storage stability results are based on the difference between the softening point values of top and bottom temperature.

The storage stability test was conducted to figure out if the t binder stable or unstable during the storage period. The difference between the top temperature and bottom temperature must not exceed 2.2 °C. Figure 5 shows the storage stability result based on the difference between the softening point values of top and bottom temperatures. It is observed in Figure 5, that all binders in this study were stable and suitable for storage without significant separation.

As seen in Figure 5, with a small amount of CR less than 1.5% of the binder, it cannot affect the storage stability of the binder. In the same way, WFO with 2.7 and 4.6% (by weight of base binder), it not affects the storage stability of the binder. In contrast, with the addition of an enormous amount of CR in bitumen. It can negatively affect the storage stability of the binder [25]. The binders consist of 25, and 40% RAPB have the lowest storage stability values. It can be related to again process during the service life of the asphalt pavement [38]. Consequently, all binders in this study are less than 2.2 °C. Therefore, all binders are storage stable.

#### 3.2.5. Bleeding

**Figure 6:**
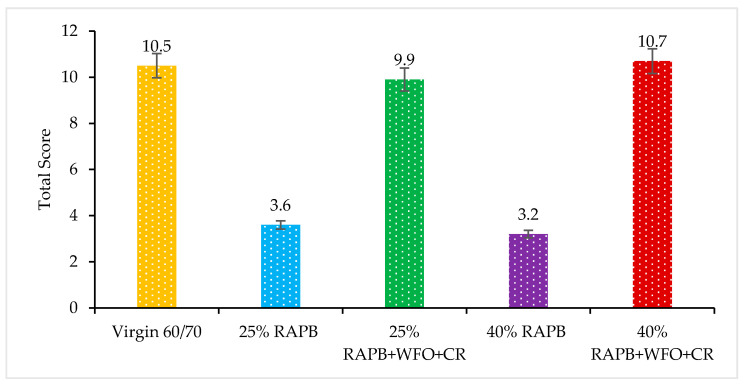
Overall score of the bleeding result for all asphalt binders.

A high temperature can cause a bitumen film on the s pavement surface, known as bleeding; the bleeding affects the skid resistance of the asphalt pavement particularly in wet conditions. Furthermore, the bleeding happened when the oily molecules in bitumen expand due to the high temperature and lack of air voids, which tend the fatty particles to up the pavement’s surface. As a result, the pavement’s surface will be shiny and glassy and may lead to a lack of problems for the drivers due to the low skid resistance of the pavement surfaces [6]. Figure 6 demonstrates the total score of the bleeding result for all binders.

Based on the outcome in Figure 6, the binders with 25 and 40% of RAPB content have the lowest values compared with the other binders. It is related to the decreased oily particles in the aged binder during the service life of the asphalt pavement. The binders with 25 and 40% of RAPB content give low bleeding values with 3.6 and 3.2, respectively. According to Zaumanis et al. [6], the massive percentage of rejuvenators can increase bleeding. The binders with 25 and 40% of RAPB content incorporating the combination of WFO and CR significance impact the aged binder. In addition, the binders with 25 and 40% of RAPB content incorporating the combination of the WFO and CR give similar bleeding values with 9.9 and 10.7, respectively, compared to the virgin bitumen with 10.5.

Consequently, with a reasonable percentage of the WFO to rejuvenate all physical properties of the aged binder. It needs a small amount of CR because it absorbs a small amount of the available oily molecules in bitumen, which could give the ability to reduce the bleeding in the binder. Besides, the CR modified provides a balance to the physical characteristics of the binder.

A paired t-test was used to compare the significant difference of virgin bitumen with RAPB and RAPB. Table 7 displays the significant differences of binders for the bleeding results. It was found that there were statistically significant differences between the virgin and 25 and 40% of RAPB binders with (*p* ≤ 0.05). Meanwhile, there is no significant difference between the virgin bitumen with 25% of RAPB content with WFO and CR and 40% of RAPB content with WFO and CR with *p*.= 0.104 and *p* = 0.816, respectively.

#### 3.2.6. Viscosity

**Figure 7:**
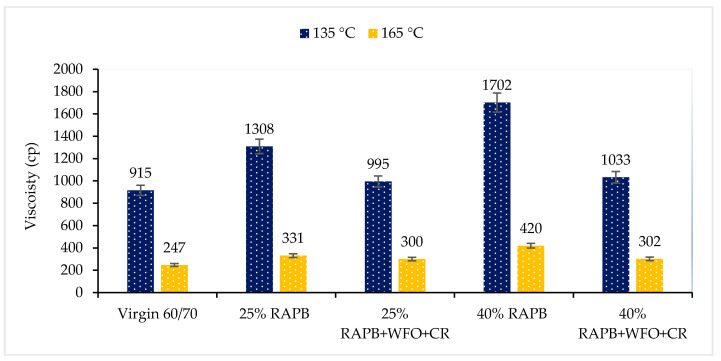
Viscosity results for two different temperatures.

The rotational viscosity test was determined at 135 and 165 °C to evaluate the workability for bituminous binders. Figure 7 demonstrates the viscosity result of the binders for different combinations of WFO and CR on the aged binders. The increase in test temperature from 135 to 165 °C leads to a decrement in the viscosity result of all the binders. However, when it reduces the mixing and compacting temperatures, less energy and cost are required to construct asphalt roads with considering the workability of the binder too. The viscosity of the binder increases with the increase in the service lifetime and aging period of the asphalt mixture [25].

The result in Figure 7 demonstrates that binders with 25 and 40% of RAPB content have high viscosity result values. This is related to the evaporation of the oily particles during the service life of the asphalt pavement, which reflects the high viscosity in the binders with 25 and 40% of RAPB content. Also, the high viscosity values in the aged binder can lead to a lack of workability in the asphalt mixture. The lack of workability causes a loss in cohesion and adhesion between the aggregate and the binder [19]. As discussed by Sengoz and Isikyakar. [41], the rise in the viscosity is not convenient because the binder with a massive viscosity degree demands a higher laying, compaction, and mixing heat, implying excessive energy consumption.

On the other hand, both binders with 25 and 40% of RAPB content incorporating the combination of WFO and CR have a significant result similar to virgin bitumen because WFO decreased the viscosity of the aged binder. Besides, the CR has a slight impact on viscosity. It is one of the most important reasons to choose the CR as modified, which adjusts the stiffness with a minor effect on the viscosity of the binder. This finding is linked to the addition of a light element, which may reduce molecular interaction in aged binder [42]. Incorporating the WFO with CR on the aged binder can decrease the compaction and mixing temperatures concerning the dosage of the WFO. In conclusion, the addition of the WFO reduced the viscosity of the binder in different temperatures, which agreed with the previous study done by Chen et al. [43].

The summary of analysis using paired t-test comparisons between virgin bitumen and RAPB for viscosity result is shown in Table 8. It can be noticed that all binder content only RAPB provides significant differences when compared with virgin bitumen with (*p* ≤ 0.05). On the other hand, there were no statistically significant differences between virgin bitumen and 25 and 40% of RAPB content with WFO and CR with *p* = 0.120 and *p* = 0.078, respectively.

Furthermore, according to the viscosity results at 165 °C, the test results were compared using paired t-test statistical analysis to compare the significant difference level of virgin bitumen and RAPBs. Table 9 shows the significant differences in the viscosity results at 165 °C. It found that there is a significant difference between virgin bitumen and 25% and 40% of RAPB content with (*p* ≤ 0.05). On the other hand, there is no significant difference between the virgin asphalt mixture and the recycling asphalt mixture (*p* > 0.05).

#### 3.2.7. Ductility

**Figure 8:**
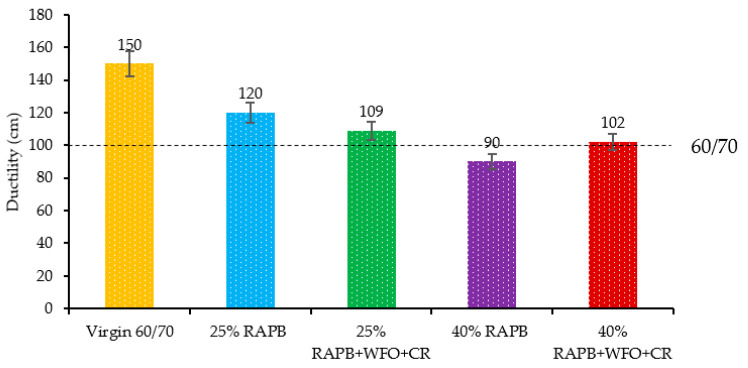
Ductility result for all binders.

Figure 8 presents the outcome of the ductility test for the different binders. It is observed in Figure 8, the addition of WFO increases the ductility result values. Also, the increase in the ductility value led to improved crack resistance, low-temperature susceptibility, and the performance of bituminous binder in general [16]. The binders with 25% and 40% of RAPB content combine virgin and aged binder. The binder with 25% RAPB content was passed the limitation with 109 cm.

In contrast, the binder with 40% of RAPB content does not pass the minimum ductility test requirement of 90cm. Thus, the aged binder, when mixed with a virgin binder, decreased the ductility values. Along with this, the decrement in ductility of binder content 40% of RAPB is strongly noticeable with a high amount of aged binder, which led to a lack of flexibility and cracked resistance [37].

On the other hand, the binders with 25% and 40% of RAPB content incorporating the combination of WFO and CR passed the ductility requirements and gave acceptable result values with 120 and 102 cm, respectively. Therefore, the ductility result for combining the WFO and CR passes the Superpave specification, which gives a result value of more than 100 cm. Table 10 presents the significant differences of group mean between virgin and RAP and RAPB for ductility result. Table 10 illustrations statistically significant differences between virgin bitumen and 25 and 40% of RAPB content binder with (*p* ≤ 0.05). In contrast, there is no significant difference between the virgin bitumen and 25 and 40% of RAP content with WFO and CR with *p* = 0.104 and *p* = 0.080, respectively.

#### 3.2.8. Loss of Heating

**Figure 9:**
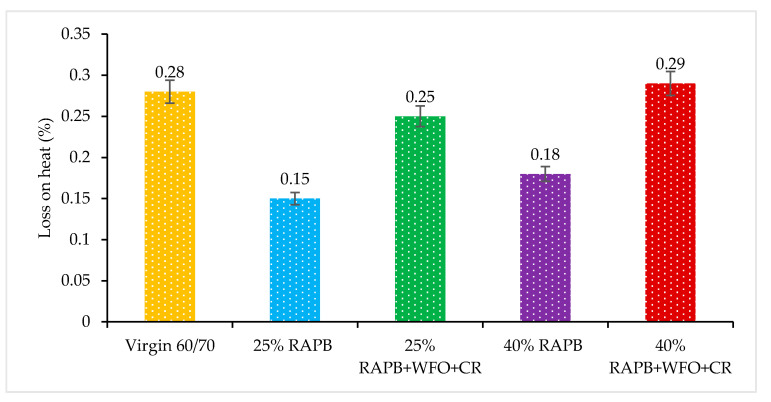
Loss of heating results for all binders.

Loss on the heat of experiment conducted using Rolling Thin Film Oven (RTFO). The loss on the heat test is used to determine the mass loss of the bituminous binder after exposure to the high temperature and pressure. At high temperatures, impurity and particles that include in the binder will volatile and release into the air. As a result, the bituminous binder mass will reduce. The typical range of heat loss is 0.05–0.5% and the maximum percent of loss on heating is 1.0% [6]. Figure 9 illustrates the loss of heating for all binders.

Based on the result in Figure 9, all the binders within the specification and the loss in mass of all binders consider as acceptable values. Also, all the binder’s values were similar to the virgin bitumen or less than it. The binders with 25% and 40% of RAPB content have the lowest loss on heating result values. Due to this, the aged binder already loses the impurity and particles during the service life of asphalt pavement. Also, the loss on heating values in the binder with 25% and 40% of RAPB content slightly lower compared to the other binders, which was consistent with previous research [44]. The binders with 25% and 40% of RAPB content incorporating a combination of WFO and CR have comparable result values to the virgin bitumen. Based on the results, all binders in this study were within the specification of the loss on heating test requirements, which is between 0.05 to 0.5%.

A paired t-test was used to compare the significant difference of virgin bitumen with RAP and RAPB. Table 11 displays the significant differences of asphalt binders for the loss on heating results. It was found that there were statistically significant differences between the virgin and 25% and 40% of RAPB content binders with (*p* ≤ 0.05). Meanwhile, there is no significant difference between the virgin bitumen with 25% of RAPB content with WFO and CR and 40% of RAPB content with WFO and CR with *p*.= 0.184 and *p* = 0.622, respectively.

#### 3.2.9. Fourier Transform Infrared Spectroscopy (FTIR)

**Figure 10:**
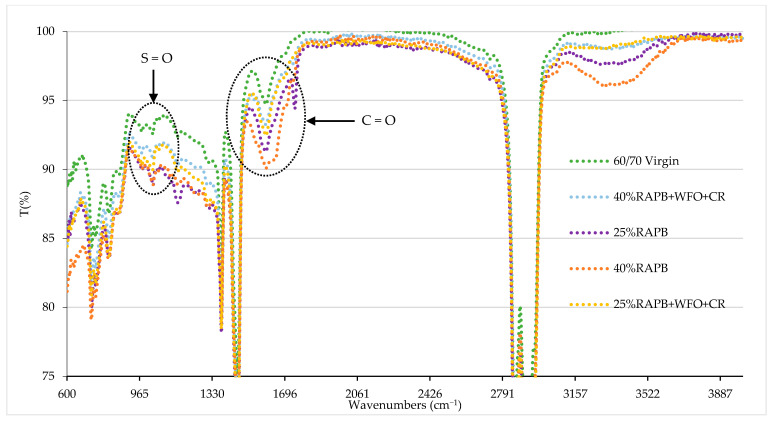
Results of FTIR spectra of the virgin, RAP, and recycled binders.

In this research, the FTIR test was conducted to measure the variation in properties of unaged and short-term-aged binders. Figure 10 displays the results of the FTIR spectra of the virgin, RAP, and modified binders. In Figure 10, absorption peaks near 2700–3000 cm^−1^ can be noted, which corresponds to O–H vibrations in a carboxylic acid. The absorption peaks at 2930 cm^−1^ are correlated with the C-H stretching of alkane. The peaks at 1456 and 1376 cm^−1^ correspond to the bending of C-H, indicating the functional groups of C-H₂, and CH₃, respectively. The small bands in the range of 660–950 cm^−1^ are typical of C–H vibrations of benzene bands as mentioned in previous studies [45,46].

The bands in the range of 1492-1700 cm^−1^ were applied as an indicator to assess the relative oxidation of virgin, RAP, and modified binders. According to a previous study done by Tayh et al. [47], the peaks at 1700 cm^−1^ represent carbonyl peaks C=O and increase with an increased amount of RAP dosage. The RAP with WFO and CR decreased the oxidation level at the band 1700 cm^−1^. It can be related to the addition of the WFO, which agrees with the previous study by Poulikakos et al. [48]. As can be observed in Figure 10, the binders containing 25 and 40% of RAPB have a higher sulfoxide group dosage than the virgin bitumen because the RAPB was exposed to the aging process, which increased the level of oxidation. These findings are similar to [49], who conducted a study on the impact of aging on the RAP with bio-based oil as a rejuvenator. In short, the addendum of the RAPB leads to an increase in the sulfoxide and carbonyl band [50]. The addition of WFO with CR reduces the sulfoxide group’s values, indicating a reasonable resistance to oxidation in the binder throughout the sulfoxide bond. It can be concluded that the addendum of WFO softens the RAPB due to the excessive content of oxygen in WFO. The rejuvenation mechanisms of hybrid rejuvenated asphalts fundamentally concentrate only on the physical mixing [42]. Another study concluded that the oxygenated groups (C=O and S=O) reduced when the aged binder was blended with a rejuvenator, indicating the potential to rejuvenate the aged binder [51].

**Figure 11:**
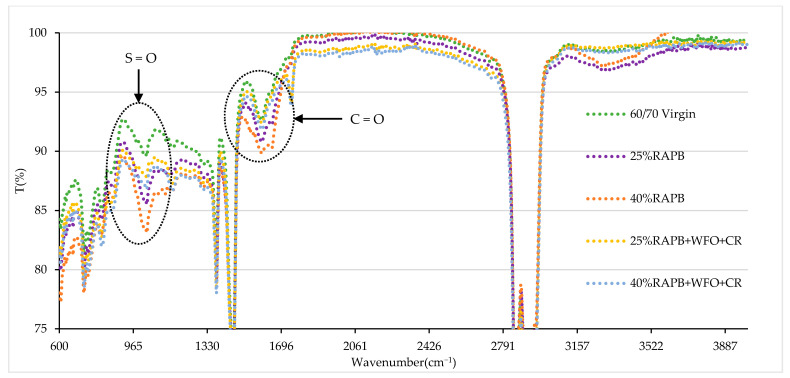
Results of FTIR spectra of the virgin, RAP, and recycled binders under short-term aging conditions.

The RTFO was conducted to confirm no change in the chemical groups after the aging process, representing the pressure and heat of bitumen during the mixing process. Figure 11 shows the results of a chemical group of the virgin, RAP, and recycled binders after short-term aging. The concentration of carbonyl, alcohol, ether, and ester slightly decreased, indicating the loss of lightweight particles for binders with 25 and 40% RAP. Meanwhile, both the virgin and modified binders had slightly increased concentrations of the carbonyl bond and sharply increased sulfoxide content due to the oxidation process of RTFO. The results of short-term aging and its effects on the chemical groups are similar to the previous study done by Tayh et al. [47].

### 3.3. Rheological Properties of Bituminous Binders

#### 3.3.1. Complex Modulus

**Figure 12:**
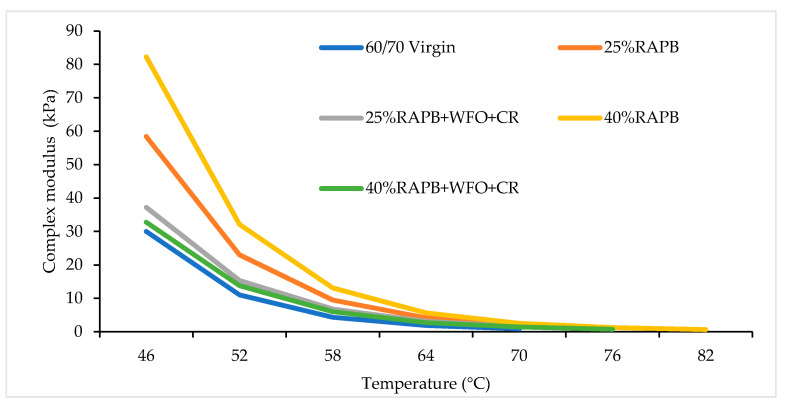
Complex modulus results of all binders.

A binder with a large complex modulus (G*) has a higher resistance to deformation [43]. Figure 12 shows the complex modulus obtained for the binders. It is observed that the addition of 40% of RAP in asphalt increased the G* value and stiffened the binder. The high G* with 40% of RAP is due to the aging process during the asphalt service life. The binder with 25% RAP has a significant G* value in comparison with the virgin bitumen. The binders with 25% and 40% RAP content show G* values with 58.4 and 82.2 kPa at 46 °C, respectively. In contrast, the binders with 25% and 40% RAP content incorporating the combination of CR with WFO represent G* values similar to the virgin bitumen. Also, the binders with 25% and 40% RAP content incorporating the combination of WFO and CR give a G* value of 37.2 and 32.7 kPa at 46 °C, respectively. There is a decrement in the G* values when the temperature increases from 46 °C to the failure temperature. This finding agrees with the previous study done by Singh-Ackbarali et al. [52], implying that the use of frying oil reduces the stiffness of the binder. 

The results were analyzed using a paired t-test to compare the significant differences of all binders with virgin bitumen. Table 12 presents a comparison of the significant differences between the virgin bitumen and binders content RAPB and RAPB. There is a significant difference between the virgin bitumen and binder contain 25% and 40% RAP with *p* = 0.022 and *p* = 0.011. Meanwhile, there is no significant difference between the virgin bitumen and RAPB with WFO and CR (*p* > 0.05). The addition of WFO and CR reduced the G* value when it was added to the RAPB.

#### 3.3.2. Phase Angle

**Figure 13:**
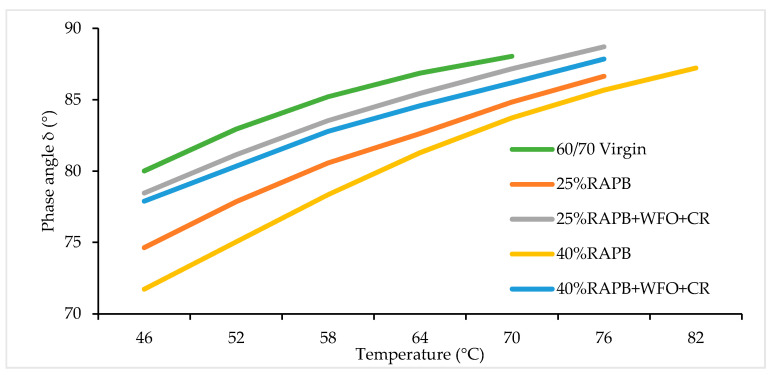
Phase angle result of the virgin, RAPB, and recycled asphalt binders.

The phase angle (*δ°*) represents the elastic degree of bituminous binder, and its values range from 0° to 90° [53]. The dynamic shear rheometer reading was taken at a control frequency of 1.59 Hz (10 rad/s), representing a vehicle speed of 90 km/h [13]. Figure 13 illustrates the *δ°* result of the virgin, RAP, and RAPB. As seen in Figure 13, there is an increment in the *δ°* due to the increase in temperature from 46 °C to failure temperature, which agrees with previous research done by Dony et al. [13]. The binders with 25 and 40% RAP content have the lowest *δ°* values compared with the virgin and modified binders. The binders with 25% and 40% of RAP content have the *δ°* values of 74.6° and 71.7° at 46 °C, respectively. On the other hand, the binders with 25% and 40% RAP content incorporating the combination of WFO and CR exhibited comparable *δ°* values to the virgin bitumen. The binders with 25% and 40% RAP content incorporating the combination of WFO and CR have the *δ°* values of 78.5° and 77.9° at 46 °C, respectively. This outcome agrees with many previous types of research, such as the study conducted by Maharaj et al. [54].

Table 13 presents the significant differences of group mean between virgin and RAPB and RAPB for *δ°* result. Table 13, it is shown that there were statistically significant differences between virgin bitumen and 25 and 40% of RAPB content binder with (*p* ≤ 0.05). The analysis showed that minor significant differences in the binder contain 25% RAPB because the *p*-value was *p* = 0.040, which is close to 0.05. In contrast, there is no significant difference between the virgin bitumen and 25 and 40% of RAP content with WFO and CR with *p* = 0.500, and *p* = 0.219, respectively.

#### 3.3.3. Rutting Resistance Parameter

**Figure 14:**
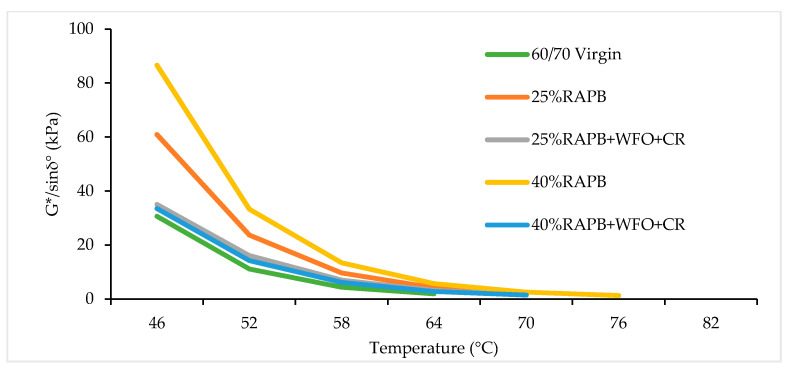
Rutting parameter result of virgin, virgin, RAP, and recycled binders.

The key to enhance the high-temperature resistance to deformation of bituminous binder is to assess the high-temperature performance of the binder. Consequently, rheology has become a valuable instrument in describing the binder’s high-temperature performance [55]. G*/sin *δ* is the rutting resistance criterion that should be more than 1.0 kPa for the unaged sample according to Superpave standard. Binders with high G*/sin *δ* value but small flow deformation at high temperatures are considered as high rutting resistance, the rutting resistance parameter can directly reflect the resistance to deformation at high temperatures [43]. Figure 14 shows the result of the rutting parameter for the virgin, RAP, and RAPB. It was observed that increasing the temperature test led to lower rutting resistance for all binders. Figure 14 illustrates that binders with 25% and 40% RAP content have high rutting resistance compared with the control sample at 46 °C, which is consistent with the outcomes of the penetration and viscosity. The binders with 25% and 40% of RAP content give rutting resistance values with 60.87 and 86.61 kPa at 46 °C, respectively. Figure 14 also shows that the binders with 25% and 40% RAP content incorporating WFO and CR combined improved the rutting resistance compared to the control sample. The same outcome was obtained by Sun et al. [37], who concluded that the addendum of oil reduced some of the higher-molecular-mass chains in the binder, which made the binder less stiff. In addition, the binders with 25% and 40% RAP content incorporating the combination of WFO and CR give rutting resistance values of 35.02 and 33.47 kPa at 46 °C, respectively.

The failure or critical temperature is represented as the temperature obtained for the ratio of the G*/sin *δ* parameter, which is more than 1.0 kPa for the unaged status according to the Superpave requirements [56]. The failure temperature increased by 6 and 12 °C when 25% RAP and 40% RAP were added, respectively, compared to the control sample. On the other hand, the combination of the WFO and CR reduced the failure temperature by 6 °C in comparison with the aged binder. The binder with 25% RAP content incorporating WFO/CR has the same failure temperature as the virgin bitumen (64°C). The failure temperature was reduced from 76 to 70 °C in the combination of WFO/CR binder with 40% RAP content and this result is comparable with the control sample.

The summary of analysis using paired t-test comparisons between virgin bitumen and RAPB and RAPB for rutting resistance result is shown in Table 14. It can be observed that all binders content only RAPB provides significant differences when compared with virgin bitumen with (*p* ≤ 0.05). On the other hand, there were no statistically significant differences between virgin bitumen and 25 and 40% of RAPB content with WFO and CR with *p* = 0.120 and *p* = 0.113, respectively.

## 4. Conclusions

The primary purpose of this study was to obtain proper workability and restore the RAPB properties by adding WFO and CR in the RAP binder. Adding a high quantity of WFO is essential to recover all the physical characteristics of the RAPB, and the softness issue can be controlled by adding a small quantity of CR with only a slight effect on the workability. The combination of CR and WFO in RAPB shows similar penetration, softening point, and loss on heating results compared to virgin bitumen. Meanwhile, the combination of both materials also improves the workability of the RAPB and storage stability. The FTIR result of all binders shows no significant change due to the non-chemical reaction between the additive materials. After RTFO, the oxidation level of both virgin and recycled binder increases, and a considerable increase in the sulfoxide peak for all the binders are observed. Furthermore, the combination of WFO/CR gives a similar failure temperature result compared to the virgin bitumen and subsequently improves the rutting resistance. In summary, a blend of WFO/CR may successfully be used to recycle RAPB, particularly at 25 and 40%.

## Figures and Tables

**Table 1:** Physical, rheological, and chemical properties tests of the bituminous binder.

Binder Tests	Test Methods	Specifications
Penetration at 25 °C (PEN)	ASTM D5	60–70
Softening Point (°C)	ASTM D36	48–52
Ductility at 25 °C (cm)	ASTM D113	Min. 100
Viscosity at 135 °C, 165 °C (cp)	AASHTO T201	-
Storage stability (°C)	-	<2.2
Bleeding	-	0–21
Penetration index	-	−2 to 2
Loss on heating (%)	AASHTO T240	Max. 1.00
Fourier transforms infrared spectroscopy	-	-
Dynamic shear rheometer	AASHTO T315	-

**Table 2:** Physical properties for various resources of WFO.

Physical Property	Result Value		
	Source 1	Source 2	Source 3
Specific gravity	0.942	0.995	0.983
pH	3.5	4.5	4.5
Moisture content (%)	0.13	0.29	0.21
Flashpoint (°C)	220.3	110	139
Viscosity at 30 °C (cp)	57.5	45.9	49.2
Loss on heat (wt%)	0.14	0.94	0.77

**Table 3:** Fatty acids of the waste frying oil.

Fatty Acid	Formula	System Name	Content (%)
Margaric	C₁₇H₃₄O₂	heptadecanoic acid	6.88
Arachidic	C₂₀H₄₀O₂	Eicosene, (E)-acid	0.22
Linolenic	C₁₈H₃₀O₂	6-Octadecenoic acid	6.94
Linoleic	C₁₈H₃₂O₂	9,12-Octadecadienoic acid	11.38
Oleic	C₁₈H₃₄O₂	9-Octadecenoic acid	54.81
Palmitic	C₁₆H₃₂O₂	Hexadecanoic acid	2.51
Palmitoleic	C₁₆H₃₀O₂	n-Hexadecanoic acid	8.92
Stearic	C₁₈H₃₆O₂	Octadecenoic acid	3.43
Myristic	C₁₄H₂₈O₂	Tetracosahexaene	0.98
Other acids with a content of less than 0.1			0.85
Total			100

**Table 4:** Comparison of significant difference of group means for the penetration result.

Compare Group Mean	Mean	Std. Deviation	Std. Error Mean	95% Confidence Interval of the Difference		Sig. (2-Tailed)
				Lower	Upper	
Virgin 60/70—25% RAP	35.16	1.4046	0.628	33.41592	36.90408	0.000
Virgin 60/70—25% RAP+WFO+CR	−0.84	1.0899	0.487	−2.19336	0.51336	0.160
Virgin 60/70—40% RAP	−0.86	0.7829	0.350	−1.83215	0.11215	0.000
Virgin 60/70—40% RAP+WFO+CR	39.652	0.3654	0.163	39.19829	40.10571	0.070

**Table 5:** Comparison of significant difference of group means for the softening point result.

Compare Group Mean	Mean	Std. Deviation	Std. Error Mean	95% Confidence Interval of the Difference		Sig. (2-Tailed)
				Lower	Upper	
Virgin 60/70—25% RAP	−5.75	0.35355	0.25	−8.92655	−2.57345	0.028
Virgin 60/70—25% RAP+WFO+CR	0.75	1.06066	0.75	−8.77965	10.27965	0.500
Virgin 60/70—40% RAP	−8.5	0.70711	0.5	−14.8531	−2.1469	0.037
Virgin 60/70—40% RAP+WFO+CR	1.25	1.76777	1.25	−14.6328	17.13276	0.500

**Table 6:** Comparison of significant difference of group means for the PI result.

Compare Group Mean	Mean	Std. Deviation	Std. Error Mean	95% Confidence Interval of the Difference		Sig. (2-Tailed)
				Lower	Upper	
Virgin 60/70—25% RAP	0.375	0.00707	0.005	0.31147	0.43853	0.008
Virgin 60/70—25% RAP+WFO+CR	0.315	0.03536	0.025	−0.00266	0.63266	0.050
Virgin 60/70—40% RAP	0.265	0.00707	0.005	0.20147	0.32853	0.012
Virgin 60/70—40% RAP+WFO+CR	0.375	0.0495	0.035	−0.06972	0.81972	0.059

**Table 7:** Comparison of significant difference of group means for the bleeding result.

Compare Group Mean	Mean	Std. Deviation	Std. Error Mean	95% Confidence Interval of the Difference		Sig. (2-Tailed)
				Lower	Upper	
Virgin 60/70—25% RAP	6.86667	0.37859	0.21858	5.92619	7.80715	0.001
Virgin 60/70—25% RAP+WFO+CR	0.66667	0.40415	0.23333	−0.33729	1.67062	0.104
Virgin 60/70—40% RAP	7.46667	0.25166	0.1453	6.84151	8.09183	0.000
Virgin 60/70—40% RAP+WFO+CR	−0.1	0.65574	0.37859	−1.72896	1.52896	0.816

**Table 8:** Comparison of significant difference of group means for the viscosity result at 135 °C.

Compare Group Mean	Mean	Std. Deviation	Std. Error Mean	95% Confidence Interval of the Difference		Sig. (2-Tailed)
				Lower	Upper	
Virgin 60/70—25% RAP	−393.2	3.70135	1.65529	−397.796	−388.604	0.000
Virgin 60/70—25% RAP+WFO+CR	−3.94	4.46856	1.9984	−9.48845	1.60845	0.120
Virgin 60/70—40% RAP	−788.2	4.20714	1.88149	−793.424	−782.976	0.000
Virgin 60/70—40% RAP+WFO+CR	−18.5	17.56417	7.85493	−40.3088	3.3088	0.078

**Table 9:** Comparison of significant difference of group means for the viscosity result at 165 °C.

Compare Group Mean	Mean	Std. Deviation	Std. Error Mean	95% Confidence Interval of the Difference		Sig. (2-Tailed)
				Lower	Upper	
Virgin 60/70—25% RAP	−87.3333	7.57188	4.37163	−106.143	−68.5238	0.002
Virgin 60/70—25% RAP+WFO+CR	−33.1667	34.41051	19.86692	−118.647	52.31378	0.237
Virgin 60/70—40% RAP	−173	4.5483	2.3094	−182.937	−163.063	0.000
Virgin 60/70—40% RAP+WFO+CR	−35.5	30.3768	17.53805	−110.96	39.96016	0.180

**Table 10:** Comparison of significant difference of group means for the ductility result.

Compare Group Mean	Mean	Std. Deviation	Std. Error Mean	95% Confidence Interval of the Difference		Sig. (2-Tailed)
				Lower	Upper	
Virgin 60/70—25% RAP	29.33333	10.01665	5.78312	4.45059	54.21608	0.037
Virgin 60/70—25% RAP+WFO+CR	40.86667	24.80027	14.31844	−20.7406	102.474	0.104
Virgin 60/70—40% RAP	60	10	5.7735	35.15862	84.84138	0.009
Virgin 60/70—40% RAP+WFO+CR	48	25	14.43376	−14.1034	110.1034	0.080

**Table 11:** Comparison of significant difference of group means for the loss on the heating result.

Compare Group Mean	Mean	Std. Deviation	Std. Error Mean	95% Confidence Interval of the Difference		Sig. (2-Tailed)
				Lower	Upper	
Virgin 60/70—25% RAP	0.13	0.03	0.01732	0.05548	0.20452	0.017
Virgin 60/70—25% RAP+WFO+CR	0.02	0.01732	0.01	−0.02303	0.06303	0.184
Virgin 60/70—40% RAP	0.11	0.01732	0.01	0.06697	0.15303	0.008
Virgin 60/70—40% RAP+WFO+CR	−0.01	0.03	0.01732	−0.08452	0.06452	0.622

**Table 12:** Comparison of significant difference of group means for the G* result at different temperatures.

Compare Group Mean	Mean	Std. Deviation	Std. Error Mean	95% Confidence Interval of the Difference		Sig. (2-Tailed)
				Lower	Upper	
Virgin 60/70—25% RAP	−27.45	1.3435	0.95	−39.5209	−15.3791	0.022
Virgin 60/70—25% RAP+WFO+CR	−4.355	4.03758	2.855	−40.6312	31.92121	0.369
Virgin 60/70—40% RAP	−51.37	1.23037	0.87	−62.4244	−40.3156	0.011
Virgin 60/70—40% RAP+WFO+CR	−1.635	1.60513	1.135	−16.0565	12.78654	0.386

**Table 13:** Comparison of significant difference of group means for the *δ°* at different temperatures.

Compare Group mean	Mean	Std. Deviation	Std. Error Mean	95% Confidence Interval of the Difference		Sig. (2-Tailed)
				Lower	Upper	
Virgin 60/70—25% RAP	5.735	0.51619	0.365	1.09724	10.37276	0.040
Virgin 60/70—25% RAP+WFO+CR	0.775	1.09602	0.775	−9.07231	10.62231	0.500
Virgin 60/70—40% RAP	8.145	0.20506	0.145	6.3026	9.9874	0.011
Virgin 60/70—40% RAP+WFO+CR	1.56	0.79196	0.56	−5.55547	8.67547	0.219

**Table 14:** Comparison of significant difference of group means for the G*/sin *δ* at different temperatures.

Compare Group mean	Mean	Std. Deviation	Std. Error Mean	95% Confidence Interval of the Difference		Sig. (2-Tailed)
				Lower	Upper	
Virgin 60/70—25% RAP	−37.085	2.5668	1.815	−60.1468	−14.0232	0.031
Virgin 60/70—25% RAP+WFO+CR	−3.71	1.00409	0.71	−12.7314	5.31141	0.120
Virgin 60/70—40% RAP	−51.505	2.12839	1.505	−70.6278	−32.3822	0.019
Virgin 60/70—40% RAP+WFO+CR	−2.435	0.61518	0.435	−7.9622	3.0922	0.113

## Data Availability

All data used in this research can be provided upon request.
